# Driving ability and predictors for driving performance in Multiple Sclerosis: A systematic review

**DOI:** 10.3389/fneur.2022.1056411

**Published:** 2022-11-30

**Authors:** Susan Seddiq Zai, Christoph Heesen, Carsten Buhmann, Roshan das Nair, Jana Pöttgen

**Affiliations:** ^1^Institute of Neuroimmunology and Multiple Sclerosis (INIMS), Center for Molecular Neurobiology, University Medical Center Hamburg-Eppendorf, Hamburg, Germany; ^2^Department of Neurology, University Medical Center Hamburg-Eppendorf, Hamburg, Germany; ^3^Health Division, SINTEF, Trondheim, Norway; ^4^School of Medicine, University of Nottingham, Nottingham, United Kingdom

**Keywords:** multiple sclerosis (MS), driving, on-road driving, driving simulator, cognition

## Abstract

**Objective:**

To provide an overview of the evidence on driving ability in persons with multiple sclerosis (PwMS), specifically to (i) study the impact of MS impairment on driving ability and (ii) evaluate predictors for driving performance in MS.

**Methods:**

To identify relevant studies, different electronic databases were screened in accordance with PRISMA guidelines; this includes reference lists of review articles, primary studies, and trial registers for protocols. Furthermore, experts in the field were contacted. Two reviewers independently screened titles, abstracts, and full-texts to identify relevant articles targeting driving in people with MS that investigated driving-related issues with a formal driving assessment (defined as either an on-road driving assessment; or naturalistic driving in a car equipped with video cameras to record the driving; or a driving simulator with a steering wheel, a brake pedal, and an accelerator).

**Results:**

Twenty-four publications, with 15 unique samples (*n* = 806 PwMS), were identified. To assess driving ability, on-road tests (14 papers) and driving simulators (10 papers) were used. All studies showed moderate to high study quality in the CASP assessment. About 6 to 38% of PwMS failed the on-road tests, showing difficulties in different areas of driving. Similarly, PwMS showed several problems in driving simulations. Cognitive and visual impairment appeared to most impact driving ability, but the evidence was insufficient and inconsistent.

**Conclusion:**

There is an urgent need for more research and standardized guidelines for clinicians as one in five PwMS might not be able to drive safely. On-road tests may be the gold standard in assessing driving ability, but on-road protocols are heterogeneous and not infallible. Driving simulators assess driving ability in a standardized way, but without standardized routes and driving outcomes, comparability between studies is difficult. Different aspects, such as cognitive impairment or vision problems, impact driving ability negatively and should be taken into consideration when making decisions about recommending driving cessation.

**Systematic review registration:**

Identifier [10.17605/OSF.IO/WTG9J].

## Introduction

Multiple sclerosis (MS) is a chronic neurodegenerative disease that is one of the most common neurological diseases in young adults (1). MS frequently causes severe disabilities related to vision, mobility in the upper and lower limbs, and cognition (e.g., slower information processing, impaired attentional functions, and visuospatial deficits) (2–5). Three out of every four people with MS (PwMS) have gait dysfunction and limited mobility (6, 7), with walking disabilities, especially leading to patients' reliance on driving. However, driving is a complex task that involves many cognitive, visual, and motor domains, and impairment in these domains has an impact on driving ability and driving safety (8–10). MS is characterized by visual impairment and cognitive deficits, which are major risk factors for drivers (11–19).

Research shows that PwMS are more likely to be involved in automobile accidents than people without MS (20), make more mistakes while driving (21), and are 3.4 times more likely to visit the emergency department because of automobile accidents (20). Therefore, a number of studies have explored the association between MS and driving in the last decades, but despite considerable evidence for reduced driving performance, there are no clear guidelines regarding fitness for driving in MS. Little is known about MS and problems with specific driving parameters, such as control of speed, tracking stability, and recognition of dangerous situations (22). A review by Krasniuk et al. (22) found only two tests to predict driving ability in MS: The Stroke Driver Screening Assessment (23) and Useful Field of View test (24, 25) (UFOV).

Measures to assess driving ability and evaluation thresholds differ across countries, regions, and are largely not evidence-based (26, 27). A review by Fragoso et al. (28) showed five different approaches to measuring driving ability in MS: (1) measuring driving ability through self-report; (2) reporting crashes and traffic violations through government or institutional data; (3) assessing crash-risk indices through computer-based assessment; (4) observing driving performance through driving simulator assessment; and (5) evaluating fitness to drive through on-road assessment. They conclude, however, that there is no specific literature on driving abilities in PwMS that assists in creating legislation (28). Even if clinical standards for suspending driving licenses exist in practice, it is frequently an arbitrary decision based on experiences and unspecific decisions of healthcare professionals. However, driving is important for maintaining independence for PwMS, and driving cessation is related to isolation, depression, and functional decline (29, 30). Therefore, there is an urgent need not only for clinicians but also for lawgivers and traffic authority personnel to have fair and reliable regulations and guidelines to evaluate driving abilities in PwMS.

Thus, the aim of this systematic review was to study (i) the impact of MS impairment on driving ability and (ii) to identify predictors for driving performance in MS. Additionally, we provide an overview of the available evidence on driving ability to determine whether the findings are sufficient to help develop standardized guidelines in future and to locate possible gaps in the literature to improve future research and clinical practice if not.

## Methods

### Search strategy

This systematic review was planned, conducted, and reported in accordance with PRISMA guidelines (www.prismastatement.org) and the protocol was registered with OSF Registry (10.17605/OSF.IO/WTG9J). A comprehensive search was performed on the Cochrane Library, Ovid MEDLINE, PsycInfo, Embase, and CINAHL databases to locate relevant studies up to 1 January 2022. We also screened reference lists of review articles and primary studies, checked trial registers (clinicaltrialsregister.eu, rialregister.nl, and isrctn.com) for protocols, and contacted experts in the field to identify further published or unpublished studies. The search strategy was developed by the author/co-author team and was guided by an expert on Systematic Reviews (AR and JuP).

This review is part of a project to review driving ability in people with neurodegenerative disorders. Therefore, the initial database search terms incorporated several neurological diseases, but for this study, only data about PwMS were extracted. The search terms included MeSH terms as well as the following 14 key terms: multiple sclerosis, Parkinson's disease, Alzheimer's disease, dementia, neurodegenerative disorder, Huntington, drive, accidents, traffic, simulation, on-road, car, automobile, and vehicle, as well as different spellings of those words. The search strategy for this review is shown in Supplementary material 1. Two database searches were conducted, the initial search (up to April 2020) and an update search (up to January 2022). In the second database search, the keywords for the other diseases beside MS were excluded.

### Study inclusion criteria

Articles that met the following criteria for inclusion were selected for full-text review and analysis: (1) original research article published in a peer-reviewed journal; (2) English or German language; (3) human studies; (3) full-text available; (4) included drivers with a diagnosis of MS; (5) investigating driving-related issues with a formal driving assessment (formal driving assessment was defined as (a) an on-road driving assessment; (b) naturalistic driving in a car equipped with video cameras to tape the driving; or (c) a driving simulator. The driving simulator had to have a steering wheel, a brake pedal, and an accelerator to be considered); (6) used quantitative methods for data collection and reported quantitative results in the analysis. Studies were excluded if they (1) presented data in which PwMS were mixed in with other groups, or (2) were commentary articles, literature reviews, case studies, conference abstracts, and proceedings or dissertations.

### Quality assessment

We used the Critical Appraisal Skills Programme (31) (CASP) tool to determine the validity and quality of the included studies. We adapted the CASP tool slightly to enhance the validity and reliability of the quality assessment. We used the CASP Case-Control Study Checklist for the assessment. Scores ranged from 0 to 6 points with higher values indicating better quality. Two reviewers (SSZ and JP) assessed the studies separately accordingly to the CASP requirements and resolved discrepancies by discussion.

### Screening strategy and data extraction

After the database searches, we removed duplicates. Two researchers worked independently to screen the remaining titles and abstracts for inclusion (SSZ and EF). Any discrepancies raised during this process were resolved by discussion with the team. For the full-text screening stage, two researchers (SSZ and KW) worked independently to include/exclude studies using the selection criteria. Disagreements were resolved by discussion and consensus. Information extracted from included studies were: (1) study design (for longitudinal studies only pre-test assessments were included); (2) methodological considerations; (3) inclusion and exclusion criteria; (4) participant characteristics and demographics; (5) comparison of control group characteristics; (6) results of specific outcome measures; and (7) key findings. Data extraction was executed by two independent authors (SSZ and JP) and cross-checked.

### Evidence synthesis

Only a narrative review (without meta-analyses) was undertaken due to the vastly different outcome measures, sample characteristics, and study designs of the included studies. The existing evidence is reported according to the different assessments for driving (on-road driving vs. driving in a driving simulator). MS-related disease factors influencing the assessed driving ability are summarized based on the available studies reporting driving assessment outcomes.

## Results

### Paper selection, characteristics of selected studies, and participants included

**Figure 1:**
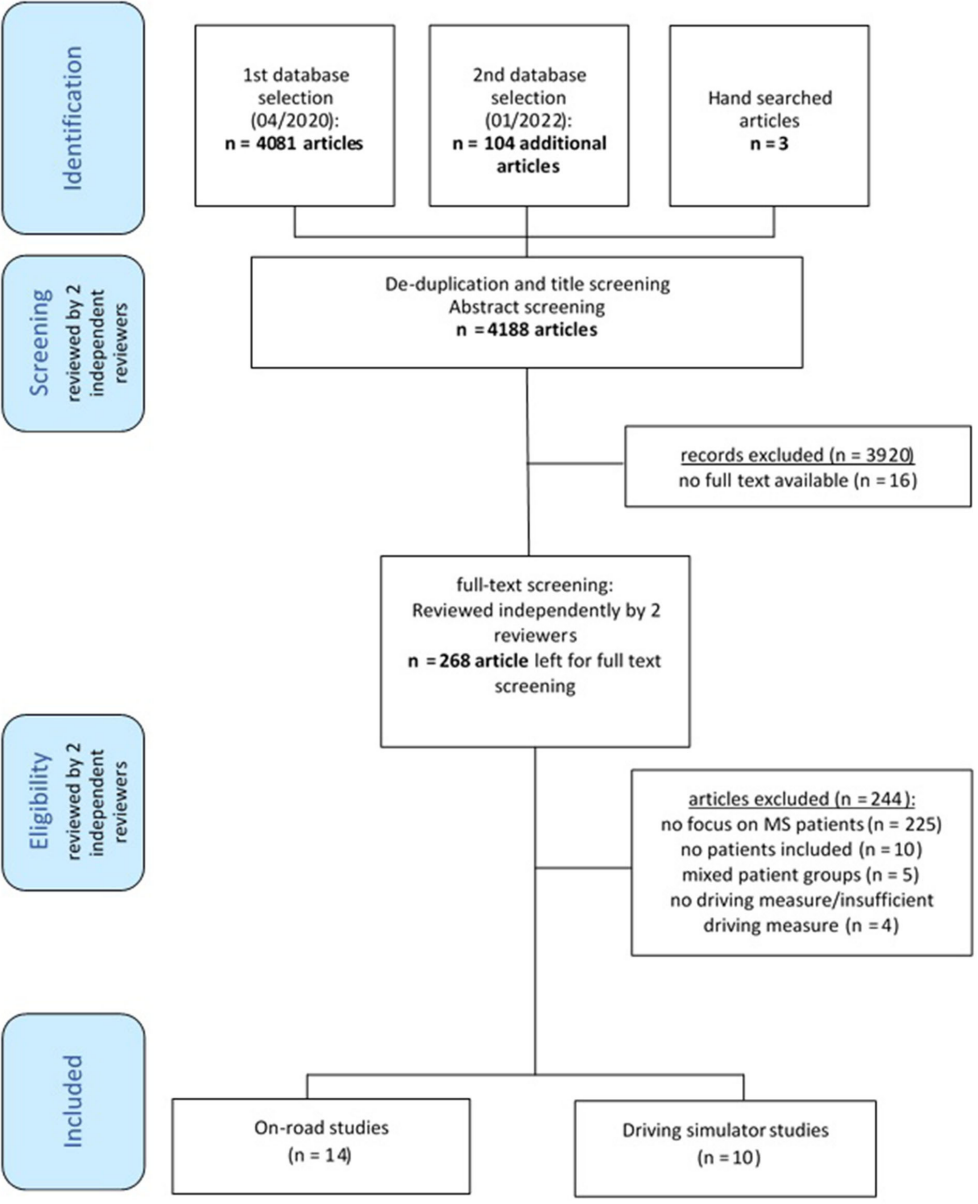
Flow diagram of all included studies.

The database search yielded 4,081 papers (Figure 1). We included 24 papers for this review (11–19, 32–46). All included studies were published in English.

The papers covered 15 different/unique samples (some papers reported data with the same study sample) with 806 PwMS (n = 553 women; 69 %) and 280 non-MS controls (n = 146 women; 52 %). The same samples reported in more than one paper are marked with ^*n^ in the citation. Fourteen studies assessed driving ability with an on-road test, while 10 studies used a driving simulator.

Demographic and disease-specific data of all included studies are shown in Supplementary material 2. Most studies included small to moderate sample sizes: on-road studies ranged from 30 (13^*2^) to 218 (43) PwMS (mean: 65 PwMS included per study), while driving simulator studies ranged from 11 (36) to 38 (39^*3^, 40^*3^) PwMS (mean: 25 PwMS included per study). The age of the PwMS ranged between 35.6 years (mean, SD 8.3) (17) and 55 years (median, Q1–3: 50–59) (35). Most PwMS had relapsing-remitting MS (n = 439), 25 were classified as SPMS, 29 had PPMS, and 13 were reported as unknown. Four studies did not report the MS subtype (n = 300) (18, 41, 43, 44). The disability level of the participants was reported *via* EDSS in almost all samples except for three (16^*5^, 41, 43, 46^*5^), and ranged from 1.95 (mean, SD 0.91) to 6.00 (median, range 3.0–7.5). One study used the ambulation index to assess disability (16^*5^), and the other two studies did not assess disability level (41, 43). Six hundred nineteen PwMS (n = 429 women; 69 %) were assessed via on-road driving test (see Table 1) and 187 PwMS (n = 124 women; 66 %) in a driving simulator (see Table 2). Nine studies used healthy participants as controls (17, 18, 34–36, 39^*3^, 40^*3^, 45^*4^, 46^*5^). One study included older volunteers as controls (13^*2^). The studies were conducted in the US (*n* = 12), Canada (*n* = 7), Belgium (*n* = 2), France (*n* = 1), United Kingdom (*n* = 1), and Germany (*n* = 1).

**Table 1:** Main results in on-road studies (x^*n*^ = paper report the same sample).

**Authors (year)**	**Study design**	**Driving experience**	**Assessments for driving performance**	**Main findings**
Akinwuntan et al. (32^*1^) (MS only)	Cross-sectional, not consecutively recruited	Pass: 28 years (median, Q1-3: 19–36) Fail: 30 years (median, Q1-3:25–36)	Standardized on-road evaluated with a 16-item checklist (approaching traffic signs, checking blind spots, speeding, braking, lane keeping, lane changing, staying in center of lane, following, signalling, right of way) (a total score >44 = passed)	34 (77%) passed the on-road driving test, 10 (23%) failed
Akinwuntan et al. (11^*1^) (MS only)				
Akinwuntan et al. (33^*1^) (MS only)	Longitudinal, not consecutively recruited	TG: 27 years (median, Q1-3: 20–35) CG: 36 years (median, Q1-3: 18–38)		More than 80% of participants in both groups passed the test before training commenced. 5 of 7 participants in the training group who initially failed the road test passed at post-training, the participant in the CG who failed at pre-test also failed in the post-test (n.s.).
Akinwuntan et al. (12) (MS only)	cross-sectional, not consecutively recruited	31.02 years (mean, SD 9.16)	Standardized on-road evaluated with the Test Ride for Investigating Practical fitness-to-drive (TRIP)	99 (84%) passed the on-road driving test, 19 (16%) failed.
Classen et al. (13^*2^) (MS vs. volunteers)	cross-sectional, not consecutively recruited	N/A	Standardized UWO on-road course + GRS (pass, pass with recommendation, fail with remediation, or fail)	24 passed (82%), 5 failed (17%), 1 (1%) was excluded because of vision problems. PwMS who failed (vs. passed) made significantly more adjustment to stimuli (*p* = 0.02), gap acceptance (*p* = 0.03), and total number of driving errors (*p* = 0.04). Differences between MS and volunteers are not evaluated and reported in this
				Review because of the differences in the demographics and measures
Krasniuk et al. (37^*2^) (MS only)				29 (78%) passed, 8 failed (22%), Adjustment to stimuli and gap acceptance errors were more common in PwMS who failed
Krasniuk et al. (15^*2^) (MS only)				28 (80%) passed, 7 (20%) failed PwMS who failed (vs. passed) made significantly more lane maintenance (*p =* 0.02) and speed regulation errors (*p =* 0.03)
Krasniuk et al. (38^*2^) (MS only)				No differences between MS and HC, Adjustment to stimuli and gap acceptance errors increased the odds of PwMS failing the on-road test
Morrow et al. (42^*2^) (MS only)				22% were deemed unfit to drive
Devos et al. (14) (MS only)	Cross-sectional, not consecutively recruited (only MS)	31.06 years (mean, SD 8.87, range: 10–49). Annual mileage−1,000 miles/y: 2.8 (median, Q1-Q3: 1.04–10.00, range 0.2–55)	Standardized on-road evaluated with the TRIP	Most PwMS showed submaximal performance in TRIP scores
Lincoln and Radford (41) (MS only)	On-road, consecutively recruited from people referred for assessment at the Derby Regional Mobility center (UK), (only MS)	23.8 years (mean, SD 9.07, range 8–48) time since last having driven a car 0 months (median, range 0–72)	Standardized on-road evaluated by an approved driving instructor + Nottingham Neurological Driving Assessment	21 passed the on-road test, 7 failed. 6 PwMS were counted as “fail” for not being able to participate on the on-road test (limb problems, eye problems)
Ranchet et al. (43) (MS only)	On-road, consecutively recruited at the Center for Evaluation of Fitness to drive and Car Adaptations of the Belgian Road Safety Institute for patients that needed a medical clearance, (only MS)	Pass: 33 years (median, Q1-Q3: 25–39) Fail: 37 years (median, Q1-Q3: 21–41)	Standardized road-test by either an occupational or physical therapist certified to conduct practical fitness-to-drive evaluations	14 (6%) failed, 204 (94%) passed
Schultheis et al. (45^*4^) (MS vs. HC)	On-road, not consecutively recruited	MS: 24.8 years (mean, SD 7.56). HC: 17.8 years (mean, SD 9.29)	BTW driving evaluation, administered by a certified driver rehab specialist + 33-item checklist, Schultheis 2010 + DMV composite score based on violations /collision in the past 5 years	53 MS passed (80%), 12 MS (20%) classified borderline
Schultheis et al. (19^*4^) (MS only)				2 (3%) did not complete all predictor variables. 52 passed (81%), 12 (19%) failed

MS, multiple sclerosis; Q1–3 = first quartile to third quartile; Pass, passing the on-road test; Q1–3 = first quartile to third quartile; Fail, failing the on-road test; TG, training group; CG, control group; n.s., not significant; SD, standard deviation; TRIP, Test Ride for Investigating Practical fitness-to-drive; N/A, not available; UWO, University of Western Ontario's; GRS, Global Rating Score; PwMS, persons with multiple sclerosis; HC, healthy controls; BTW, behind-the-wheel; DMV, Department of Motor Vehicles.

**Table 2:** Main results of driving simulator studies.

**Authors (year)**	**Study design**	**Driving** **experience**	**Assessments for driving** **performance**	**Main findings**
Devos et al. (34) (MS vs. HC)	cross-sectional	N/A	Standardized route with on a driving simulator (stationary mock-up car, with STISIM Drive^®^ software). Response time, accuracy, number of accidents, traffic tickets, speed variability, SDLP and TTC were computer generated. DA symbols were randomly projected in the side mirrors.	No difference in the driving task between PwMS and HC. PwMS responded significantly slower (*p =* 0.001) and less accurately (*p* < 0.001) on the DA task compared to the healthy controls.
Devos et al. (35) (MS vs. HC)	cross-sectional	N/A	Portable driving simulator powered on STISIM Drive^®^ (Time to completion, distance over speed limit, distance out of lane, defined as the percentage of total distance drivers crossed the center line or the road edge.	No difference in the driving simulator outcomes between PwMS and HC.
Harand et al. (36) (MS vs. HC)	cross-sectional, not consecutively recruited	>2 years + 5,000 km/year	SIM2INRETS fixed-base driving simulator equipped with an ARCHISIM object database. Approximately 60 min. drive, 3 conditions (the monotonous condition, the divided attention condition, and the urban driving condition) (measures: LP, mean speed, lane crossing, SDLP, SDS, errors and omissions, response time, accidents and collisions)	Patients showed less effectiveness for the SDLP than HC in the monotonous driving condition (*p* < 0.05), for the driving simulation with DA condition (*p* ≤ 0.01), and for the standard deviation of fixed goal speed (*p* < 0.01). Patients made significantly more errors and omissions for visual cues than HC (*p* < 0.01). There was no significant difference between groups concerning the urban driving condition and the number of accidents and collisions, and other driving related variables.
Kotterba et al. (17) (MS vs. HC)	cross-sectional	N/A, ≥2 years	Driving Simulator, model C.A.R.^®^ Simulator (Dr. Ing. R. Foerst, Gummersbach) outcomes: number of accidents and concentration faults (Driving with headlights switched off at night time; Driving with headlights switched on in the daytime; Disregarding the speed limit; Driving with dimmed headlights; Tracking error–turning too far to the right or left side of the road, touching the kerbstones or the opposite lane; Not using the flash of the headlights; Disregarding traffic light; Disregarding the right of way).	Compared to controls PwMS had more accidents (p < 0.001) and concentration faults (e.g., disregarding the speed limit, tracking error, disregarding traffic light) (*p* < 0.01). No differences in distance/60 min.
Krasniuk et al. (39^*3^) (MS vs. HC)	cross-sectional, not consecutively recruited	MS: 25.2 years (mean, SD 10.8); HC: 23.7 years (mean, SD 10.7)	CDS 200 driving simulator (operational, tactical, and strategic driving maneuvers during two scripted events (i.e. traffic light changes colors and pedestrian walks out in front of driver) and a navigational driving task.	PwMS made more adjustment to stimuli errors in the tactical driving maneuver than HC (*p* ≤ 0.05), lower response time in the pedestrian event (*p* ≤ 0.05), no differences in the traffic light event (mean speed; stopped response; failed to stop), nor in reaction time in the pedestrian event, no differences in the navigational driving task (correct decision; incorrect decision).
Krasniuk et al. (40^*3^) (MS vs. HC)			operational, tactical, and strategic driving maneuvers during four scripted hazardous events (i.e., car pulls out, traffic light changes colors, pedestrian walks in front, and vehicle cuts across lane) and a navigational driving task that occurred in 1.5-minute intervals in suburban or urban environments.	PwMS had a shorter time to collision (*p =* 0.001) and a faster mean speed (*p =* 0.04) which increased the odds of experiencing a rear-end collision.
Marcotte et al. (18) (MS vs. HC)	cross-sectional, not consecutively recruited	N/A Annual mileage (MS: 6,293 km/year, Q1–3: 3,521–9,938; HC: 9,569 km/year, Q1–3: 5.344–17.197)	Driving simulator STISIM drive^®^ software (outcomes: lane position, speed, car following, response to divided attention stimuli, SDLP). The primary outcomes were as follows: (1) coherence between the participant and lead cars (a general correlation [0 −1] of the participant's ability to accurately track the speed variations of the lead car); (2) time delay (or the reaction time to changes in the lead car's speed); and (3) modulus (the average ratio of the following vehicle's speed to the lead vehicle's speed). The third outcome (the modulus) was used to measure the degree to which participants overcompensate (> 1) or undercompensate (< 1) their separation distance from the lead car at any point in the time series.	The MS group drove significantly faster than HC on the lane-tacking condition (*p =* 0.03) and had a greater variability in speed maintenance (*p =* 0.002). They also had greater deviation in lane position than HC (*p =* 0.001). PwMS showed more difficulty than HC in tracking the movements of the lead car (*p* < 0.001) and were slower to respond to changes in speed (*p =* 0.074). No differences in the degree to which the groups over- or undercompensated the distance from the lead car.
Raphail et al. (44) (MS only)	cross-sectional, not consecutively recruited	N/A. At least 1 year	Virtual reality driving simulator (outcomes: variability in speed and variability in lane position)	Increased severity on the MSFC was correlated with greater variability in lane position (*p =* 0.01) but not to variability in driving speed.
Schultheis et al. (46^*5^) (MS only)	cross-sectional, not consecutively recruited	Years of driving: no CI: 28.5 (mean, SEM 2.1), with CI: 22.7 (mean, SEM 2.8),	Neurocognitive Driving (outcomes: Test (NDT)–NDT-latency / NDT-errors)	The MS (with CI) group performed significantly worse than both the MS (no CI) and HC groups in the latency to perform several driving-specific functions on the NDT (*p* < 0.001), but no overall group differences were observed
		HC: 26.7 (mean, SEM 2.0)		in actual errors on the NDT. No significant differences between HC and MS (no CI).
Shawaryn et al. (16^*5^) (MS only)		25.7 (mean, SEM 1.7, Range 4–38) Average no. of days driving weekly: 5.8 (mean, SEM 0.4, range 0.25–7.0)		The overall MSFC score correlated significantly with the NDT latency score

MS, multiple sclerosis; HC, healthy controls; N/A, not available; STISIM, Systems Technology; Inc. Simulation; SDLP, standard deviation of lateral position; TTC, time to collision; DA, divided attention; PwMS, persons with multiple sclerosis; LP, mean lateral position of the vehicle; SDS, standard deviation of fixed-goal speed; C.A.R., computer-aided risk; SD, standard deviation; CDS, clinical driving simulator; Q1–3 = first quartile to third quartile; MSFC, multiple sclerosis functional composite; CI, cognitive impairment; SEM, standard error of the mean; NDT, Neurocognitive Driving Test; no., number.

### Study quality assessment

Results of the CASP assessments are shown in Supplementary material 3. Quality ratings for the included studies ranged from 3/6 to two studies rated with 6/6 points (39^*3^, 40^*3^). No study was excluded for poor quality. Heterogeneity in samples, data collection, and measurement of identified variables was present between studies. All participants were screened before being included in the studies. Ten studies recruited a control group but only seven delivered a comparable number of matched controls (18, 34–36, 39^*3^, 40^*3^, 46^*5^). All studies performed the driving assessment (on-road or in a driving simulator) accurately (e.g., driving instructors blinded to group allocation) to minimize bias. Thirteen studies considered confounding factors appropriately (11^*1^, 12, 14, 16^*5^, 19^*4^, 32^*1^, 33^*1^, 39^*3^, 40^*3^, 41, 43, 45^*4^, 46^*5^). The quality of the results was evaluated as acceptable only in 11 studies (11^*1^, 12, 14, 15^*2^, 19^*4^, 37^*2^, 38^*2^, 39^*3^, 40^*3^, 42^*2^, 45^*4^).

### Outcomes of included studies

#### On-road driving assessment

Fourteen studies (seven unique samples) used on-road driving to assess driving ability (Table 1). Twelve of the 14 on-road driving studies showed that 6 to 38 % of PwMS failed the on-road driving test (11^*1^–13^*2^, 15^*2^, 19^4^, 32^*1^, 33^*1^, 37^*2^, 41, 42^*2^, 43, 45^4^), with 10 out of 12 studies suggesting that between 17 and 23% of PwMS are unfit to drive (11^*1^–13^*2^, 15^*2^, 19^*4^, 32^*1^, 33^*1^, 37^*2^, 42^*2^, 45^*4^). Lincoln & Radford (41) reported the highest fail rate (38%, 13 out of 34 PwMS), while Ranchet et al. (43) observed a low fail rate (6 %). Akinwuntan et al. (12) had eight PwMS who initially failed the on-road assessment retake the test (seven of them trained in a driving simulator prior to the second on-road test). Five of the participants passed the second on-road assessment, while two PwMS in the training group and the one without training failed. Devos et al. (14) did not differentiate between “pass” and “fail” in their study but found that most PwMS demonstrated sub-maximal performance, with participants reaching a total on-road score of 184.15 (mean, SD 13.48) out of 196 in the Test Ride for Investigating Practical fitness-to-drive (TRIP). Adjustment to stimuli (i.e., responding to critical roadway information) and gap acceptance errors (i.e., errors in merging into a street traffic stream because the gap between two cars was miscalculated) was identified as indicators of failing the on-road test, because more PwMS who failed the on-road test had problems in those areas (13^*2^, 37^*2^, 38^*2^). Lane maintenance and speed regulation errors were also identified as predictors for failing the on-road test (15^*2^).

#### Assessment in a driving simulator

Ten studies (eight unique samples) assessed driving ability by a driving simulation (16^*5^–18, 34–36, 39^*3^, 40^*3^, 44, 46^*5^). Driving simulator measures and results are shown in Table 2. Eight out of 10 studies compared the driving ability of PwMS to healthy controls (HCs) (17, 18, 34–36, 39^*3^, 40^*3^, 46^*5^). Unlike the on-road studies, driving simulator studies did not evaluate whether PwMS passed or failed the driving assessment. Harand et al. (36) let PwMS drive in three different conditions: monotonous driving condition, divided attention (DA) condition, and urban driving condition (driving along an urban road with other cars). They found that PwMS showed significantly more difficulty in the standard deviation of lateral position (SDLP, i.e., a measure of road tracking errors, “weaving” of the car) than HC in the monotonous driving condition, as well as for the standard deviation of speed. They also had more difficulties driving in the DA condition than HC. No significant differences between groups were found in the urban driving condition, or in the number of accidents and collisions. Similarly, Marcotte et al. (18) found that PwMS had significantly greater SDLP than HC, drove significantly faster on the lane-tacking condition, and had greater variability in speed maintenance. Persons with multiple sclerosis also showed more difficulty in tracking the movements of the lead car and were slower to respond to changes in speed compared to HC. The groups did not differ in the degree to which they over- or under-compensated the distance from the lead car. Kotterba et al. (17) found that PwMS made more accidents compared to controls, and had more concentration faults (described as disregarding the speed limit, tracking errors, or disregarding traffic lights). There was no difference in distance driven in the allotted timeframe (60 min) between the two groups. Krasniuk et al. (39^*3^) used operational, tactical, and strategic driving maneuvers during two scripted events (i.e., traffic light changes colors and pedestrian walks out in front of the driver) and a navigational driving task to assess driving ability. PwMS made more adjustments to stimuli errors in the tactical driving maneuver than HC and had a slower response time in the pedestrian event, but they did not differ in the traffic light event (i.e., the groups did not differ in mean speed and whether they stopped or failed to stop). There were no between-group differences in the navigational driving task or reaction time in the pedestrian event. In another publication, on the same sample, Krasinuk et al. (40^*3^) found that PwMS had a shorter time to collision and a faster mean speed, which increased the odds of experiencing a rear-end collision than HC.

Two studies did not find any significant differences in driving performance between PwMS and HC (34, 46^*5^). Schultheis et al. (46^*5^) compared PwMS with cognitive impairment (CI), PwMS without CI (no CI), and HC using the Neurocognitive Driving Test (47) (NDT), in which one component was a driving simulation. They found that CI-PwMS performed significantly worse than both the no CI-PwMS and HC groups in the latency to perform several driving-specific functions on the NDT. No overall group differences were observed in actual errors on the NDT. They did not find any significant differences between HC and no CI-PwMS.

#### Impact of demographic factors

**Figure 2:**
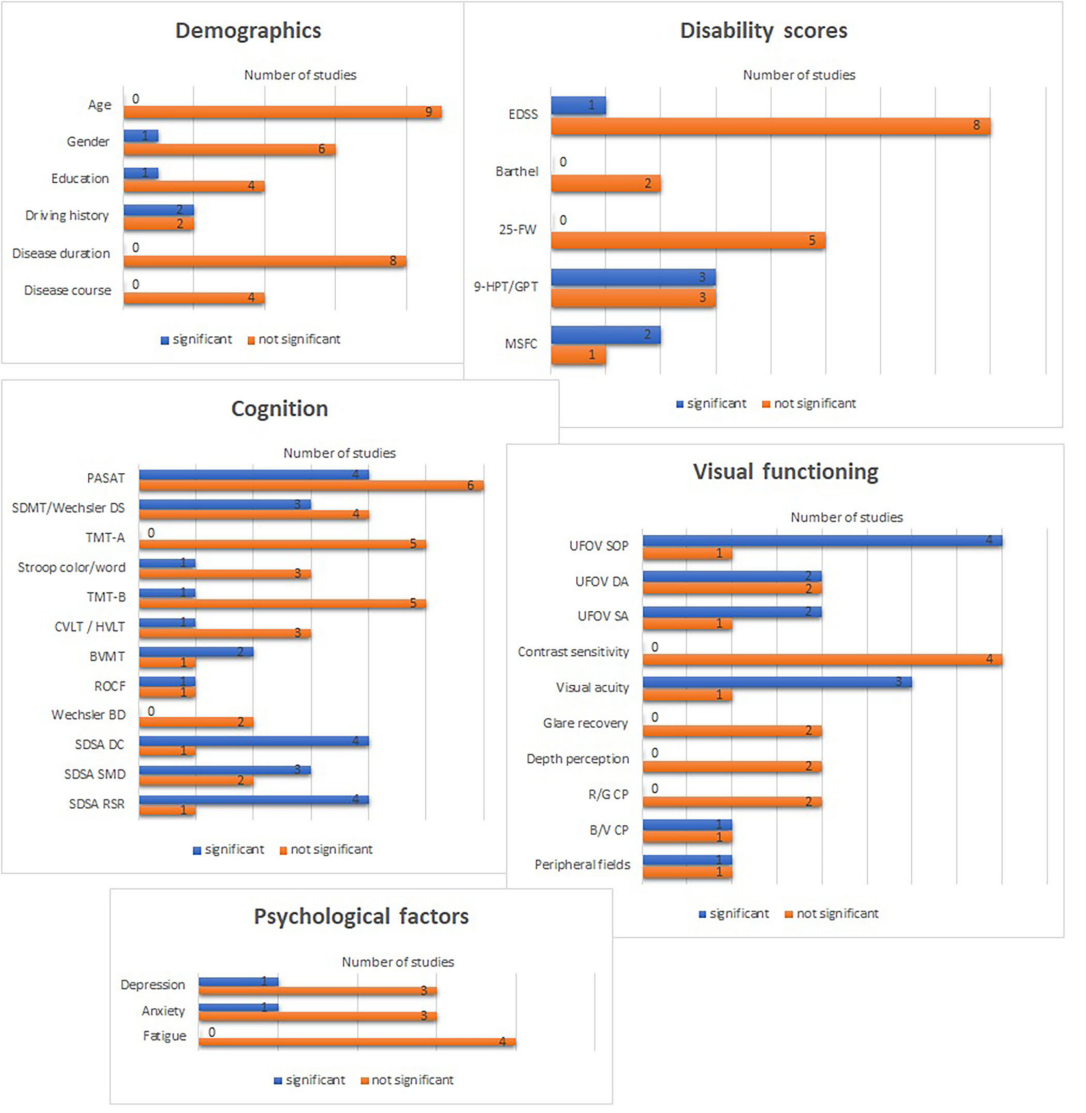
Impact of disease-related abilities on driving. EDSS, Expanded disability status scale; 25-FW, 25-foot walk-test; 9-HPT, 9 Hole peg test, GPT, Grooved pegboard test; MSFC, Multiple sclerosis functional composite; PASAT, The paced auditory serial addition test; SDMT, Symbol digit modalities test; DS, Digit symbol; TMT-A, Trail making test-A; TMT-B, Trail making test-B; CVLT, California verbal learning test; HVLT, Hopkins verbal learning test; BVMT, Brief visuospatial memory test; ROCF, Rey osterreith complex figure; BD, Block design; SDSA, Stroke driver screening assessment; DC, dot cancellation; SMD, Square matrices directions; RSR, Road sign recognition; UFOV, Useful field of view; SOP, Speed of processing; DA, Divided attention; SA, Selective attention; R/G CP, Red & green color perception; B/V CP, Blue & violet color perception.

Age as a factor that impacts driving was assessed in nine samples (11^*1^, 14, 16^*5^, 17, 32^*1^, 34, 36, 37^*2^, 41, 42^*2^, 43) (Figure 2). No study found any relation between age and driving performance. Only one (41) found gender to have an impact on driving, with more women than men failing the driving task. In five samples (11^*1^, 14, 16^*5^, 32^*1^, 36, 37^*2^, 42^*2^), the impact of education on driving performance was assessed and only one (14) found a significant correlation. Four samples examined driving history, including experience, accidents/received traffic tickets/fines (11^*1^, 14, 32^*1^, 41, 43). Devos et al. (14) found a significant negative correlation between the number of traffic tickets and driving performance. Lincoln and Radford (41) found a significant difference in PwMS who passed vs. those who failed on the on-road test and the time since participants had last driven a car. In another sample (37^*2^, 42^*2^), significantly more employed participants passed the on-road test compared to participants who were unemployed at the time of testing. Participants' country of birth and ethnicity did not have any relation to driving ability.

Disease duration (including duration since first symptom and time since onset MS) was assessed in eight studies (11^*1^, 14, 16^*5^, 32^*1^, 34, 36, 37^*2^, 41, 42^2^, 43), disease course in four (14, 34, 37^*2^, 42^*2^), and medication in one sample (37^*2^, 42^*2^), with no significant relations to driving found.

#### Impact of EDSS and motor function

In only one (19^*4^, 45^*4^) out of nine samples (11^*1^, 14, 17–19^*4^, 32^*1^, 34, 36, 37^*2^, 42^*2^, 44, 45^*4^) had a significant impact of EDSS on driving performance: patients with high EDSS failed the on-road test and performed significantly worse in driving. The Barthel index was assessed in two samples (11^*1^, 14, 32^*1^) and the 25-foot walk-Test (25FW-test) in five samples (11^*1^, 14, 17, 32^*1^, 34, 44). No significant results were found for either. The nine Hole Peg Test (9-HPT)/Grooved Pegboard Test (GPT) was used in six samples (11^*1^, 14, 17, 18, 32^*1^, 34, 44). Akinwuntan et al. (11^*1^) found significantly more impairment in hand functions in patients who failed the on-road test. Devos et al. (14) found significant correlations between 9-HPT and the driving performance score. Marcotte et al. (18) studied the impact of GPT on driving performance and found significant correlations between hand functions (dominant and non-dominant hand) with car following time delay. The multiple sclerosis functional composite (MSFC) was used in three samples (11^*1^, 16^*5^, 32^*1^, 44): one study (44) found greater variability in lane position in patients with higher impairment in MSFC and the other (16^*5^) found significant correlations between driving latency and hand functions measured by the MSFC.

#### Impact of cognition

Thirteen studies assessed cognition in relation to driving ability. We summarize the findings for each cognitive domain.

##### Attention and information processing

Ten studies used the PASAT to assess the capacity and rate of information processing (including sustained and divided attention). Only three of these found significant correlations between PASAT and driving performance measures (number of accidents, speed, latency) (16^*5^, 17, 34), and one sample found significant differences with the PASAT in PwMS who passed/failed the on-road test (11^*1^, 32^*1^). The Symbol Digit Modalities Test (SDMT)/Wechsler Digit Symbol (DS) was used in seven samples (11^*1^, 14, 15^*2^, 18, 19^*4^, 36, 39^*3^, 42^*2^). Devos et al. (14) found a significant correlation between SDMT performance and the driving protocol (Test Ride for Investigating Practical fitness-to-drive, TRIP test, with better performance in the SDMT relating to higher scores). Schultheis et al. (19^*4^) found better SDMT results in PwMS who passed the driving assessment than those who did not, and Marcotte et al. (18) found a significant correlation between the DS subtest and the Standard Deviation of Lateral Position (a measure of road tracking error) measured in a driving simulator. None found associations between Trail Making Test-A (TMT-A) and driving (11^*1^, 14, 18, 46^*5^).

##### Executive functions

The Stroop test was used in four studies (11^*1^, 12, 14, 41). Only Devos et al. (14) found significant correlations between the Stroop subtest color/word (C/W) with the TRIP. Only one (14) out of six studies (11^*1^, 14, 18, 19^*4^, 34, 46^*5^) found significant associations between Trail Making Test-B (TMT-B) and driving.

##### Learning and memory

One (18) out of four samples (15^*2^, 18, 19^*4^, 39^*3^, 42^*2^) that examined verbal learning and memory using the Hopkins Verbal Learning Test-Revised (HVLT-R)/California Verbal Learning (CVLT2) found significant correlations between lateral position and verbal learning and between car following time delay and delayed memory. Two samples (15^*2^, 39^*3^, 42^*2^) examined visuospatial learning and memory using the Brief Visuospatial Memory Test-Revised (BVMT-R). One found significant correlations between speed regulation errors and visuospatial memory (15^*2^), and one reported a significant association between impairment in immediate recall and failure on on-road driving (42^*2^).

##### Visuoconstructive functions

The Rey–Osterrieth Complex Figure (ROCF) (copy) to assess visuoconstructive abilities was used in two samples (11^*1^, 14, 32^*1^). Devos et al. (14) found significant correlations between the driving and visual-constructive functions. No significant associations between visuospatial functions (Wechsler Adult Intelligence Test [subtests BD]) and driving ability were found (11^*1^, 46^*5^).

##### Driving-related multi-domain measures

Four (11^*1^, 12, 14, 32^*1^, 41) out of five samples (11^*1^, 12, 14, 32^*1^, 34, 41) that examined the Stroke Driver Screening Assessment (SDSA) (subtests: dot cancellation [DC] [DC-time, DC-error, DC-false-positive], Square Matrices Directions [SMDs], Square Matrices Compass [SMC], road sign recognition [RSR]) found significant relations with driving abilities: three samples (11^*1^, 12, 32^*1^, 41) reported significant differences between passed vs. failed MS drivers in all subtests of the SDSA. Devos et al. (14) found significant correlations between on-road driving TRIP measures and the SDSA (DC-time, SMD, SMC, and RSR).

In total, 11 of the 13 studies which measured cognitive abilities did find a relation between cognition and driving performance.

#### Impact of psychological aspects

We found four studies examining psychological measures (depression, anxiety, fatigue) on driving (11^*1^, 14, 34, 36), but significant correlations were only found in one study between depression with time to collision (TTC), and anxiety with the simulation divided attention task (34). Fatigue did not significantly correlate with driving ability.

#### Impact of visual function

The impact of vision on driving was reported in eight studies. The UFOV (subtests: speed of processing [SOP], divided attention [DA], selective attention [SA]) was used in five studies. Two studies (11^*1^, 12) found significant differences in passed vs. failed participants in SOP and driving abilities. Classen et al. (13^*2^) reported significant correlations between SOP and gap acceptance errors. Devos et al. (14) found significant correlations between driving abilities and SOP, DA, and SA. Shawaryn et al. (16^*5^) used the UFOV to stratify PwMS into persons with low-risk and moderate/high-risk vision groups and found significantly lower driving ability scores in the low-risk group.

Four samples (13^*2^-15^*2^, 34, 43) examined the impact of acuity on driving performance: one study found a significant correlation between acuity and driving abilities (14), one reported a relation between binocular acuity and failing the on-road test (43), and one sample found a significant correlation between visual acuity and the on-road adjustment to stimuli measure (13^*2^, 15^*2^).

Four samples assessed the impact of contrast sensitivity on driving, and no significant associations were found (11^*1^, 13^*2^-15^*2^, 34). Two studies examined the impact of glare recovery on driving in MS and did not find significant associations (11^*1^, 14). They also examined red & green color perception (R/G CP) and blue & violet CP (B/V CP) (11^*1^, 14), and only one found significant differences between the driving passed vs. failed MS group in B/V CP (11^*1^). Depth perception was studied in two samples (11^*1^, 13^*2^, 15^*2^) and no significant associations were found. One study found significant correlations between the peripheral vertical measure and the on-road TRIP measure (14), but no significant associations between peripheral vision and driving ability were found in another sample (13^*2^, 15^*2^).

In total, seven of the eight studies that measured any visual aspect found a relation between at least one visual parameter and driving performance.

#### Predictors of driving performance

Different outcomes were investigated as possible predictors for driving performance (see Table 3). The outcomes used predicted driving ability with an accuracy ranging from 82 to 91%, with most analyses resulting in high specificity (79–98 %) but low sensitivity (25–80 %) (11^*1^, 12, 15^*2^, 19^*4^, 32^*1^, 38^*2^, 40^*3^, 41), except for Morrow et al. (42^*2^) who predicted pass or fail in the on-road test with 100 % sensitivity but low specificity (35.7%−53,57% depending on the combination of predictors). Commonly, the predictors tended to be related to the performance on cognitive tests (19^*4^, 32^*1^, 39^*3^, 41, 42^*2^), on predefined driving-related aspects assessed during the driving evaluation (15^*2^, 38^*2^, 40^*3^), or a combination of cognitive and visual tests (11^*1^, 12, 14). Other predictors included “having no history of driving as part of (past or current) employment” combined with cognitive tests (42^*2^), MSFC and education (16^*5^, 34), and physician recommendation on fitness to drive and binocular acuity (43). For cognitive tests, the predictors that were commonly used were SDSA (11^*1^, 32^*1^, 41), Stroop test (11^*1^, 14), and SDMT (19^*4^, 42^*2^). For vision, UFOV was found to be predictive of driving ability (11^*1^, 12).

**Table 3:** Predictors of driving ability.

**Authors (year)**	**Main findings**
Akinwuntan et al. (11^*1^)	Discriminant analyses: Performance in the four cognition tests (Stroop color, SDSA directions, compass, RSR) together with UFOV (SoP) predicted outcome of the on-road test (failed vs. passed) with 91% accuracy, 70% sensitivity, and 97% specificity
Akinwuntan et al. (32^*1^)	Discriminant analyses: prediction of Road performance in the four SDSA variables (DC, SMD, SMC, RSR (failed vs. passed) with 86% accuracy, 80% sensitivity, and 88% specificity.
Akinwuntan et al. (12)	Discriminant analyses: Performance in the four cognition tests together with UFOV (SoP) predicted outcome of the on-road test (failed vs. passed) with 82% accuracy, 42% sensitivity and 90% specificity.
Devos et al. (14)	Linear regression: TRIP was determined by a combination ROCF (*p =* 0.0002), Stroop C/W (*p =* 0.008), binocular acuity at mid-distance (*p =* 0.04), vertical visual field (*p =* 0.02) and stereopsis (*p =* 0.03)
Krasniuk et al. (15^*2^)	First model included lane maintenance errors (OR = 0.18, p =0.009, 95% CI = [0.05, 0.66]), and the second model included speed regulation errors (OR = 0.04, CI = [0.003, 0.44]), as sole predictors of pass vs. fail outcomes in PwMS. An optimal cut-point of one or more lane maintenance errors validly predicted 78 % (*p* = 0.02) of pass vs. fail outcomes, with 71 % sensitivity, 79 % specificity, and 23 % misclassification rate. An optimal cut-point of one or more speed regulation errors validly predicted 77 % (*p* = 0.03) of pass vs. fail outcomes, with 57 % sensitivity, 96 % specificity, and 11 % misclassification rate.
Krasniuk et al. (38^*2^)	Discriminant analyses: In suburban environments adjustment to stimuli and gap acceptance errors ≥4 predicted on-road outcomes with 71.4% sensitivity and 92.9% specificity. In city environments ≥2 adjustment to stimuli and gap acceptance errors predicted on-road outcomes with 57.1% sensitivity and 92.9% specificity.
Krasniuk et al. (39^*3^)	Linear regression: Traffic light event (operational): group predicted response type (*p* = 0.04). Pedestrian event (tactical): slower DA predicted adjustment to stimuli errors (*via* slower reaction time) in all participants (*p* = 0.03), deficits in immediate verbal/auditory recall, slower DA and group predicted adjustment to stimuli errors (*via* slower response time)
Krasniuk et al. (40^*3^)	Discriminant analyses: (in MS) time to collision (cut-off ≤ 1.81 seconds) predicted rear-end collisions with 85% sensitivity, 100% specificity. Mean speed (cut-off ≥7.83 meters per second) predicted rear-end collisions with 77% sensitivity and 76% specificity.
Lincoln & Radford (41)	Discriminant analyses: performance in SDSA (DC, RSR) and AMIPB (adjusted score B, design learning) predicted passed or failed with 85% sensitivity and 90% specificity
Morrow et al. (42^*2^)	Chi-square analysis: Impairment in BVMTR-IR–sensitivity 100%, specificity 35.7% Impairment in SDMT and BVMTR-IR–sensitivity, 100%, specificity 45.43%. Impairment in SDMT and BVMTR-IR with no history of driving as part of current/past employment predicted passed or failed with 100% sensitivity and 53.57% specificity.
Ranchet et al. (43)	Agreement of 88% (181/218) between physician recommendation and on-road assessor. Compared with the on-road assessor, the referring physician overestimated the fitness to drive of 11 patients and underestimated the fitness to drive of 16 patients. Full model regression analyses, including both the physician recommendation and binocular acuity, explained 24% of the total variance in the on-road decision, but binocular acuity was retained as the sole variable in the stepwise regression model, explaining 21% of the total variance in the on-road decision. Physician recommendation did not add significantly to the model (*P* > 0.05).
Schultheis et al. (19^*4^)	Linear regression: SDMT was the strongest predictor (*p =* 0.07). The model had low sensitivity (25%) but high specificity (98%) with respect to predicting failure on the BTW test.
Shawaryn et al. (16^*5^)	Regression analyses: MSFC and education did not significantly predict the NDT-errors; significant prediction of the NDT-latency score (32% of the variance [F2, 26 = 5.97, *P =* 0.007)] with the overall MSFC as the significant predictor (B = −0.58, *P =* 0.005).

SDSA, Stroke Driver Screening Assessment; RSR, road sign recognition; UFOV, useful field of view; SoP, speed of processing; DC, dot cancellation; SMD, square matrix direction; SMC, square matrix compass; TRIP, Test Ride for Investigating Practical fitness-to-drive; ROCF, The Rey–Osterrieth Complex Figure; C/W, color/word; OR, odds ratio; CI, confidence interval; PwMS, persons with multiple sclerosis; AMIPB, Adult Memory and Information Processing Battery; BVMT-R, Brief Visuospatial Memory Test-Revised; IR, immediate recall; SDMT, Symbol Digit Modalities Test; BTW, behind-the-wheel; DA, divided attention; MSFC, multiple sclerosis functional composite; NDT, Neurocognitive Driving Test.

## Discussion

Our discussion is structured around three key areas in driving and MS research. After a brief discussion of the quality of the included studies, the first key aspect addresses the assessment and evaluation of driving ability. We then synthesize the results from the individual studies to consider the evidence of association between driving ability, demographic (e.g., age, gender, education, disease duration), disease characteristics (disability scores, cognition, psychological and visual impairments), and which outcomes predicted driving ability. We have organized these findings based on (i) full agreement between studies demonstrating an association between these variables and driving ability, (ii) mixed findings, and (iii) full agreement between studies demonstrating no associations. Finally, we discuss the impact of our findings on clinical practice and recommendations for further research.

### Study quality

Most included studies were small cross-sectional studies with non-consecutively recruited participants. The studies were of satisfactory quality based on the CASP tool. Major problems identified were the selection of the control group, consideration of confounding factors, and presentation of study results (especially reporting of confidence intervals). Many studies assessed small, homogenous samples, which may have affected the results and limited the validity of study results.

### Driving on-road vs. driving simulator

Between 6 and 38% of PwMS failed the on-road test, with the highest (41) and the lowest fail rates (43) being outliers. Most individual studies reported a fail rate between 17 and 23 %. In the study with the 38% fail rate, only seven of the 13 participants considered to have not passed actually failed the on-road test, while six others were counted as unfit to drive for not being able to participate on the on-road test for other reasons (e.g., poor eyesight, dexterity, etc.) (41). Counting only those participants who took and failed their on-road test reduces the fail rate to 25%, bringing it closer to the other studies. Ranchet et al. (43), conversely, observed a very low on-road test fail rate (6%). This study had the largest sample (n=218) in our review and was the only study in which the participants were neither volunteers nor patients in a clinic setting. Participants had to attend the on-road test for legal reasons (i.e., medical clearance for driving) (43). The large sample size and mandatory participation suggest that the fail rate may resemble the MS population more closely. However, the fail rate is comparatively very low, which might be because 74% (n=162) of the participants had been evaluated before, and had already passed the driving assessment at least once, while the people who previously failed were already removed from the total sample. Also, participants who are legally required to be assessed and research volunteers might prepare for and react to the on-road test differently (e.g., the former being more stressed because of the higher stakes for them). Furthermore, even though on-road tests are considered the gold standard for assessing driving ability, there are differences between the various on-road tests because they use differing protocols. Standardizing on-road tests is possible to a certain degree, but results may still vary depending on which route is taken, or which person is evaluating driving ability, and other factors (e.g., traffic). Furthermore, for most studies, the outcome of the on-road test is passing or failing, which shows that PwMS have impaired driving ability but fail to differentiate which domains are impaired.

Assessments using driving simulators are better suited to examine which driving domains are impaired, but show conflicting results. Some studies did not find any significant differences in driving performance between PwMS and HC (11^*1^, 46^*5^), while others did (17, 18, 36). Studies that found significant differences between both groups diverged as well. Harand et al. (36) did not find a difference in the number of accidents between PwMS and HC, while Kotterba et al. (17) showed that the accident rate was significantly higher in PwMS compared to controls. This might be in part due to the small number of PwMS assessed in most of these studies, ranging from 11 to 38 participants. In addition, Kotterba et al. (17) compared 31 PwMS with 10 HC which could have led to problems with statistical power. Overall, PwMS struggled with different aspects of the driving simulator. They performed worse in the standard deviation of lateral position (18, 36), made more adjustments to stimuli errors (39^*3^, 40^*3^), had greater variability in speed maintenance (18), and had more concentration faults (17).

Driving simulator settings could be useful in clinical practice in future to determine driving ability. However, because the driving simulators used in the studies differed, it is currently difficult to compare them not only with each other but also with on-road tests. Some presented different driving scenarios (36, 39^*3^, 40^*3^), while others evaluated more than just driving ability, e.g., pre-driving questions, reaction time task, and a visual task (16^*5^, 46^*5^). More research is needed to develop a gold standard for driving in simulators, because it allows people with severe impairments to be assessed safely. However, it is unclear how closely driving in a simulator mirrors real-world driving, with studies indicating that while driving simulators are frequently used, many are not fully validated (48), while other studies claim that performance in a driving simulator reflects real-life driving ability (49) and replicates experimental road conditions that on-road tests cannot replicate (50).

### Impact of disabilities on driving ability

Different MS-related physical, cognitive, psychological, and visual impairments were found to negatively impact driving ability.

#### Consistent associations

The UFOV, which was used to study the impact of vision on driving ability, and the SDSA, which was used to analyze cognition in relation to driving, were two tests that stood out. This is in line with the review by Krasniuk et al. (22)who reported the UFOV and SDSA as the best measures to predict driving ability in MS. In our review, we found that for the UFOV, significant differences/correlations were reported in all five studies, albeit for different subtests (11^*1^, 12, 13^*2^, 14, 15^*2^, 16^*5^). Speed of processing (SoP) was especially found to impact driving ability. SoP was not associated with passing vs. failing in only one sample (13^*2^, 15^*2^), but did correlate with gap acceptance errors (13^*2^). These two papers had medium CASP ratings while the other four, with the exception of the Shawaryn et al. study (16^*5^), had higher quality, and might therefore be more reliable. Results regarding the UFOV are in line with prior research, reporting the UFOV as a potential predictor for driving ability (11^*1^, 12, 22).

The Stroke Driver Screening Assessment (SDSA) was used in five samples to assess the impact of cognition on driving ability (11^*1^, 12, 14, 32^*1^, 34, 41) and except for one study (34), all found significant results for the subtests Dot Cancellation and Road Sign Recognition. This study had medium quality in the CASP rating and a very small sample (*n* = 15) and might therefore have been underpowered. The quality of the other studies was rated higher. Both medium-quality studies did have a larger sample but lost points for not having a control group.

Visual acuity most consistently showed a significant impact on driving ability in multiple samples (three out of four) (13^*2^, 14, 15^*2^, 43). Devos et al. (34) did not find a significant relation, which again might be due to the small sample size.

#### Heterogeneous results

Most of the evidence presented in this review showed heterogeneous results. Two of the 13 studies investigating cognition did not find any relation between cognitive functioning and driving performance (39^*3^, 44), while another study did not find any differences in cognition between PwMS who passed vs. failed the driving task (15^*2^). Similarly, data was inconsistent, and in some cases contradictory, in different areas of cognition, such as learning and memory, attention and information processing, executive functions, visuo-constructive functions, and multi-domain measures. The two studies that did not find significant correlations between cognitive tests and driving ability were one of the lowest (44) and highest (39^*3^) quality-rated papers. However, the higher rated study did find that immediate verbal recall predicted adjustment to stimuli errors (39^*3^).

Physical aspects seem only partially related to driving ability, which might be due to the study samples in this review presenting PwMS with low to moderate physical disabilities, with some studies even excluding PwMS with high EDSS scores. Similarly, some visual aspects showed mixed results, with too little research to conclude. The impact of psychological aspects on driving ability was inconclusive for depression and anxiety, with each showing significant association in one out of four studies (34).

#### No associations

Demographic factors were consistently found to have little to no impact on driving ability. Similarly, MS-related factors like disease duration and disease course were not associated with driving ability. Other physical aspects showed no or only one significant association [e.g., EDSS in one out of nine samples (19^*4^, 45^*4^) (high CASP rating)]. One cognitive test that was shown to have no impact on driving ability was the TMT-A. For TMT-B, only one out of six studies (14) (high CASP rating) showed a significant association. Fatigue did not have a significant impact on driving ability.

Surprisingly, age and physical disability were not or only partially related to driving ability. This is a contrast to prior research on older drivers and other neurological diseases (such as Parkinson's disease), which show both aspects to be related to driving ability (51–54). Generally, prior research on driving ability in other cohorts and patient groups has shown that (1) age, (2) visual impairment, and (3) dementia or cognitive impairment were strong predictors for impaired driving (55, 56). While the results of the UFOV and the SDSA are in line with this, many other tests in both visual and cognitive functioning did not show convincing associations. This might be because the present cohort was relatively young, with mean ages from 36 to 55 years, homogeneous, and showed only low to moderate physical disability. Prior studies showed that older PwMS demonstrate more impairment in cognition than young and middle-aged PwMS (57) and that PwMS show a bigger decline in physical ability than in cognition in the first 10 years of MS (58). Therefore, the MS cohort could have been too young and not impaired enough to show significant differences. Another explanation could be that the tests used in the studies were not sensitive enough to show the differences.

Assessing driving ability in an older cohort of PwMS with more severe disabilities would thus be important to show whether age and MS-associated deficits become stronger predictors. Also, longitudinal studies on driving ability in PwMS are lacking but might be helpful to discover how driving ability changes over time. Driving simulators might be a safe alternative to assess the driving ability of this group without endangering them or other road users.

### Clinical and scientific impact

The present evidence shows that one out of five (~20%) PwMS might not be able to drive safely, but there is no sure way for healthcare professionals to identify them without extensive testing. Since there is a lack of validated predictive test batteries for driving ability, not only for PwMS but for other cohorts as well (49, 59), more studies with standardized tests are needed to identify new possible predictors and to give a better idea of how strongly those predictors impact driving ability. Tests such as the UFOV and the SDSA may be helpful but can also be problematic because different studies show different subtests to be related to driving ability. For clinical practice, it is important to note that impairment in one area alone does not justify driving cessation, but when combined with other forms of impairment, it may be an indicator of unfitness to drive. The findings of this review show that while standardized guidelines for driving cessations are needed, the available evidence on driving ability in PwMS is not sufficient to develop these guidelines.

Driving simulators seem to be better suited to determine which areas in driving are impaired, as the outcomes are often more differentiated than in on-road assessments, which mostly consist of passing or failing the on-road test. Most studies presented in this review were of moderate quality in the CASP assessment, but only two studies (representing one sample) had high quality. Two key aspects that were found to be in need of improvement were the inclusion of comparable control groups and larger samples. Further high-quality research in driving in PwMS is needed to enable us to make more accurate predictions of fitness to drive, and to keep PwMS and other road users safe.

## Data availability statement

The original contributions presented in the study are included in the article/Supplementary material, further inquiries can be directed to the corresponding author.

## Author contributions

CB, CH, JP, and SSZ conceived the study and developed the protocol. JP and SSZ developed the search strategies, conducted the search, selected studies for the review, extracted the study data, and drafted the manuscript. CB, CH, RdN, JP, and SSZ revised the manuscript and approved the final version. All authors contributed to the article and approved the submitted version.

## Funding

This study was sponsored via an independent grant by Merck Healthcare Germany GmbH, Weiterstadt, Germany (CrossRef Funder ID: 10.13039/100009945). The funder was not involved in the study design, collection, analysis, interpretation of data, the writing of this article or the decision to submit it for publication.

## Conflict of interest

CB has received fees for advisory board participation from UCB Pharma and Zambon, as well as lecture fees from AbbVie Pharma, BIAL Pharma, TAD Pharma, UCB Pharma, and Zambon Pharma. CH received research grants from Merck, Novartis, and speaker honoraria from Merck and Roche. JP has received funding to present lectures on fatigue from GAIA AG, for investigator-initiated studies from Merck and Celgene. RdN has received funding to present lectures on cognitive screening and rehabilitation in MS (speakers' bureau) from Merck, Novartis, and Biogen. SSZ declares that the research was conducted in the absence of any commercial or financial relationships that could be construed as a potential conflict of interest.

## Publisher's note

All claims expressed in this article are solely those of the authors and do not necessarily represent those of their affiliated organizations, or those of the publisher, the editors and the reviewers. Any product that may be evaluated in this article, or claim that may be made by its manufacturer, is not guaranteed or endorsed by the publisher.
