# Supplementary material for: Driving ability and predictors for driving performance in Multiple Sclerosis: A systematic review
Source: Front Neurol. 2022 Nov 30;13:1056411. doi: 10.3389/fneur.2022.1056411 (PMC9749487; doi:10.3389/fneur.2022.1056411)
Supplement: Supplementary file 1 [file Table_1.DOCX]

Suppl. 1 Search strategy

Search 1

1. “Multiple sclerosis”.mp. or Multiple sclerosis/
2. (“parkinson’s disease” or “parkinsons disease”).mp. or parkinson’s disease/
3. (“Alzheimer” or “Alzheimer’s disease”).mp. or Alzheimer’s disease/
4. “dementia”.mp. or dementia/
5. “neurodegenerative disease”.mp. or neurodegenerative disease/ or “neurodegenerative diseases”.mp.
6. (“neurodegenerative disorder” or “neurodegenerative disorders”).mp.
7. Neurodegeneration.mp. or neurodegeneration/
8. Huntington*.mp. or Huntington’s disease/
9. 1 or 2 or 3 or 4 or 5 or 6 or 7 or 8
10. Exp accidents, traffic/ or exp Drive/ or driv*.mp.
11. Exp simulation Training/ or simulat*.mp.
12. 10 and 11
13. “car”.mp.
14. Exp car/
15. “cars”.mp. or exp cars/
16. Automobile.mp or exp automobiles/
17. Automobiles.mp.
18. Vehicle*.mp or exp automobile driving/
19. (“on road” or “on-road” or “onroad”).mp.
20. 12 or 13 or 14 or 15 or 16 or 17 or 18 or 19
21. 9 and 20

Search 2

1. Multiple sclerosis
2. Exp accidents, traffic/ or exp Drive/ or driv*.mp.
3. Exp simulation Training/ or simulat*.mp.
4. 2 and 3
5. “car”.mp.
6. Exp car/
7. “cars”.mp. or exp cars/
8. Automobile.mp or exp automobiles/
9. Automobiles.mp.
10. Vehicle*.mp or exp automobile driving/
11. (“on road” or “on-road” or “onroad”).mp.
12. 4 or 5 or 6 or 7 or 8 or 9 or 10 or 11
13. 4 and 12
