# Supplementary material for: Driving ability and predictors for driving performance in Multiple Sclerosis: A systematic review
Source: Front Neurol. 2022 Nov 30;13:1056411. doi: 10.3389/fneur.2022.1056411 (PMC9749487; doi:10.3389/fneur.2022.1056411)
Supplement: Supplementary file 2 [file Table_2.DOCX]

Supplementary 2: Characteristics of included studies

(x^n^ = paper report the same sample population, bold author names indicate on-road study, author names in italics indicate driving simulator studies)

| **Country**  **Authors**  **(year)** | **Number of participants (n) MS/ HC** | **Sex ratio** | **Age in years MS / HC** | **EDSS** | **Disease duration** | **Disease course** | **Inclusion criteria** | **Exclusion criteria** |
| --- | --- | --- | --- | --- | --- | --- | --- | --- |
| USA  **Akinwuntan et al. (32^*1^)**  **Akinwuntan et al. (11^*1^)** | 44 MS | 37 (84%) female | Pass: 45.53 (mean, SD 11.14)  Fail: 45.10 (mean, SD 11.44) | Pass: 3 (median, Q1-3: 2-4)  Fail: 3.25 (median, Q1-3: 2-5.5) | Pass: 6.5 years (median, Q1-3: 5-13)  Fail: 3.0 years (median, Q1-3: 2-7) | RRMS only | **All 3 paper**: RRMS, age 25 – 75, valid driving license, driving experience ≥ 5 years, driving ≥ 1 time a month in previous year, minimal visual requirements to drive (binocular acuity ≥ 20/60, peripheral vision ≥ 140 degrees), no substantive cognitive impairment or dementia (MMSE ≥ 24), on stable medication/dosage, no exacerbation of symptoms 1 month prior and during study  **2013 & 2014 paper:** EDSS 1-7 | Traumatic brain injury, stroke, Parkinson’s disease, psychiatric disorders, drug/alcohol abuse |
| USA  **Akinwuntan et al. (33^*1^) ^(sub-sample)^** | 36 MS (TG, in a driving simulator)  6 MS (CG, without training) | TG: 30 (83%) female  CG: 5 (83%) female | TG: 46 (mean, SD 11)  CG: 48 (mean, SD 13) | N/A | 5 years (median, Q1-3: 3-13)  7 years (median, Q1-3: 6-13) |  |  |  |
| USA  **Akinwuntan et al. (12)** | 118 MS | 97 (82%) female | 48.05 (mean, SD 9.13) | 5 (median, Q1-3: 4-6) | 11 years (median, Q1-3: 5-16) | RRMS: 109 (92%)  PPMS: 8 (7%) | Diagnosed MS, age 25 – 75, EDSS 3-7, valid driving license, driving experience ≥ 5 years, driving ≥ 1 time a month in previous year, minimal visual requirements to drive (binocular acuity ≥ 20/60, peripheral vision ≥ 140 | Traumatic brain injury, stroke, Parkinson’s disease, psychiatric disorders, drug/alcohol abuse |
| **Country**  **Authors**  **(year)** | **Number of participants (n) MS / HC** | **Sex ratio** | **Age in years MS / HC** | **EDSS** | **Disease duration** | **Disease course** | **Inclusion criteria** | **Exclusion criteria** |
|  |  |  |  |  |  | Unknown: 1 (1%) | degrees, no substantive cognitive impairment or dementia (MMSE ≥ 24), on stable medication/dosage, no exacerbation of symptoms 1 month prior and during study |  |
| USA  *Devos et al. (34)* | 15 MS  17 HC | MS: 6 (40%) female  HC: 8 (47%) female | MS: 50 (median, Q1-Q3: 42-55)  HC: 49 (median, Q1-Q3: 26-53) | 3.5 (median, Q1-Q3: 2.5-4) | 9.25 years (median, Q1-Q3: 7 – 16) | RRMS: 8 (54%)  SPMS: 5 (33%)  PPMS: 2 (13%) | All: Age 18 years and older, valid driver’s license, driving at the moment of testing  MS: MS diagnosis, EDSS between 1 and 6 | All: binocular acuity ≤20/40  MS: clinical exacerbations or treatment with glucocorticoids in the month preceding the study, history of neurological disorders other than MS, severe muscle weakness that prevent driving a car with manual transmission  HC: history of neurological disorders |
| USA  **Devos et al. (14)** | 102 MS | 88 (86%) female | 47.91 (mean, SD 8.71, range 25-65) | 5 (median, Q1-Q3: 4-7, range: 2-7) | 9 years (median, Q1-Q3: 5-14, range: 0-37) | RRMS: 91 (89%)  PPMS: 10 (10%) | Diagnosed MS, age 25– 75, EDSS 1-7, valid driving license, driving experience ≥ 5 years, driving ≥ 1 time a month in previous year, minimal visual requirements to drive (binocular acuity ≥ 20/60, | MMSE <24, fluctuations in medication/dosage or symptom exacerbation in the month prior to the study, other neurological condition, acute psychiatric disorder, |
| **Country**  **Authors**  **(year)** | **Number of participants (n) MS / HC** | **Sex ratio** | **Age in years MS / HC** | **EDSS** | **Disease duration** | **Disease course** | **Inclusion criteria** | **Exclusion criteria** |
|  |  |  |  |  |  | Unknown: 1 (1%) | peripheral vision ≥ 140 degrees) no substantive cognitive impairment or dementia (MMSE ≥ 24), on stable medication/dosage, no exacerbation of symptoms 1 month prior and during study | drug/alcohol abuse |
| USA  *Devos et al. (35)* | 15 MS  15 HC | MS: 12 (80%) female  HC: 11 (73%) female | MS: 55 (median, Q1-3: 50 – 59)  HC: 48 (median, Q1-3: 46 – 53) | 2.5 (median, Q1-3: 2 – 3.25) (range 1-6) | 8 years (median, Q1-3: 4.5 – 15.5) | RRMS: 14 (93%)  PPMS: 1 (7%) | age 18 – 65, ability to understand instructions in English, valid driver’s license, actively driving  MS: MS diagnosis, | ocular motility problems, unresolved retina or pupillary conditions, currently taking steroids, benzodiazepines, or neuroleptics, exacerbations in the month before testing, history of any substance abuse, history of a neurological disorder other than MS |
| France  *Harand et al. (36)* | 11 MS  11 HC | MS: 10 (91%) female  HC: 7 (64%) female | MS: 41.18 (mean, SD 7.95)  HC: 40.36 (mean, SD 7.17) | 1.95 (mean, SD 0.91) | 10.3 years (mean, SD 3.62) | RRMS only | All: 30-60, right-handed, native French speakers, normal or corrected-to-normal vision, a minimum educational background of 11 years of schooling, a driver’s license (for longer than two years), regular driving (5000 km/year), absence of global cognitive | All: history of trauma, medical or psychiatric disorder that may interfere with cognition, cognitive-modifying medication |
| **Country**  **Authors**  **(year)** | **Number of participants (n) MS / HC** | **Sex ratio** | **Age in years MS / HC** | **EDSS** | **Disease duration** | **Disease course** | **Inclusion criteria** | **Exclusion criteria** |
|  |  |  |  |  |  |  | dysfunction (MATTIS DRS, ˃ 130) and absence of depressive mood (MADRS, ˂ 15), MS: clinically definite RRMS, EDSS <6, no relapses or corticosteroid therapy in the month preceding the study |  |
| Germany  *Kotterba et al. (17)* | 31 MS  10 HC | MS: 18 (58%) female  HC: 2 (20%)  female | MS: 35.6 (mean, SD 8.3)  HC: 45.1 (mean, SD 7.8) | 2.8 (mean, SD 1.4, range: 1.0 - 6.5 | 5.2 (mean, SD 4.6) | RRMS only | All: active driver, ≥ 2 years driving experience and regular driving (≥ 4 days a week)  MS: diagnosed RRMS, patients were in stable phase of disease, no psychotropic drugs | MS: a history of head injury, cerebral ischemia, and alcohol or drug abuse  HC: diseases of the central nervous system |
| Canada  **Krasniuk et al. (37^*2^)** | 37 MS | 22 (59%) female | Pass: 49.97 (mean, SD 7.27)  Fail: 52.75 (mean, SD 6.80) | Pass: 2.5 (median, range: 3)  Fail: 3 (median, range: 2) | Pass: 13.35 years (mean, SD 8.70)  Fail: 15.38 years (mean, SD 9.83) | RRMS: 22 (59%)  SPMS: 13 (35%)  PPMS: 2 (6%) | Diagnosed MS, age 18 - 59, EDSS ≤ 4, cognitive impairment in PS and EF or memory, valid driver’s license | Another diagnosis that could affect cognition (e.g. Alzheimer disease, bipolar disorder, brain injury), relapse within last 3 months, high dose of corticosteroid treatment in the month prior study, medication/ illicit drugs that cause cognitive fatigue, not comfortable on high-ways, did not meet MTO (2010) vision standards |
| Canada  **Morrow et al. (42^*2^)** | 36 MS | 22 (61%) female | 49.9 (mean, SD 7.4) | 3 (median,  range: | 13 years (mean, SD 9.0) | RRMS: 22 (61%) |  |  |
| **Country**  **Authors**  **(year)** | **Number of participants (n) MS/ HC** | **Sex ratio** | **Age in years MS / HC** | **EDSS** | **Disease duration** | **Disease course** | **Inclusion criteria** | **Exclusion criteria** |
|  |  |  |  | 1.0-5.0) |  | SPMS: 13 (36%)  PPMS: 1 (3 %) | **For Classen et al. (13^*2^):** Volunteers: age 65-75, valid driver’s license, active drivers, able to complete the questionnaires, able to drive the on-road assessment | (binocular acuity ≥ 20/50, peripheral vision ≥ 120 degrees)  **In addition only Morrow et al. (42^*2^); Krasniuk et al. (15^*2^):** evidence of major depression (BDI-FS ≥13)  **Classen (13^*2^):** Volunteers: medical advice not to drive, experiencing uncontrolled seizures, medication that caused nervous system impairment, did not meet Florida State statute or MTO (2010) (binocular acuity ≥ 20/70, with the worse eye ≥ 20/200) |
| Canada  **Krasniuk et al. (15^*2^)**  **Krasniuk et al. (38^*2^)** | 35 MS | 21 (60%) female | 50 (mean, SD 7.4) | 2.0 (median, IQR=1.5) | N/A | RRMS: 22 (63%)  progressive MS: 13 (37%) |  |  |
| Canada  **Classen et al. (13^*2^)** | 30 MS  145 volunteers | MS: 18 (60%) female  Volunteers: 62 (43%) female | MS: 50.37 (mean, SD 7.45), 52 (median,  45.75-55.25)  Volunteers: 69.90 (mean, SD 3.01), 70 (median, 67-72) | 2.5  (median) | 13 years (mean) | RRMS: 17 (57%)  SPMS: 10 (33%)  PPMS: 3 (10%) |  |  |
| Canada  *Krasniuk et al. (39^*3^)*  *Krasniuk et al. (40^*3^)* | MS: 38  HC: 21 | MS: 26 (68%) female | MS: 42.9 (mean, SD 10.3) | 2.0 (median, IQR= 1.5) | N/A | RRMS: 35 (92%)  PPMS: 3 (8%) | MS: diagnosed MS, EDSS ≤6.5  All: Age 18-59, valid driver’s license, meeting legal vision standards to drive in Ontario | Other medical, neurological, or psychiatric diagnoses; medications/illicit drugs that could affect cognitive or driving |
| **Country**  **Authors**  **(year)** | **Number of participants (n) MS/ HC** | **Sex ratio** | **Age in years MS / HC** | **EDSS** | **Disease duration** | **Disease course** | **Inclusion criteria** | **Exclusion criteria** |
|  |  | HC: 15 (71%) female | HC: 40.0 (mean, SD 9.9) | 2.0 (IQR= 1.5) |  |  | (visual acuity ≥20/50 and binocular horizontal field of view ≥120 degrees continuous) | performance; experienced relapses or had corticosteroid treatment three months prior to study; FSS >5; BDI-FS ≥14 |
| UK  **Lincoln & Radford (41)** | 34 MS | 17 (50%) female | 45.9 (mean, SD 10.4, range 24-69) | N/A | 9.3 years (mean, SD 9.82, range: 0.09-33.9) | N/A | MS, living in 50 miles radius, driving in the 2 years before referral, passed driving test prior to the onset of MS |  |
| USA  *Marcotte et al. (18)* | 17 MS  14 HC | MS: 11 (65%) female  HC: 9 (64%) female | MS:49.5 (mean, SD 7.9)  HC: 47.7 (mean, SD 11.7) | 6 (median, range 3.0 - 7.5) | N/A | N/A | All: regular drivers in the year before the study (≥ 400 km/past year)  MS: had definite or probable MS, complaints of spasticity and at least moderate increase in tone, age 18 – 65, fluent in English, on a stable dose of baclofen or tizanidine for at least 3 months preceding the study. | MS: Axis I psychiatric disorder (eg. major depressive disorder, bipolar disorder), neurologic disease other than MS, recent active substance abuse, an unstable medical problem, used benzodiazepines (to control spasticity), sedatives or high doses of analgesic medications  HC: history of a central nervous system disorder that might affect |
| **Country**  **Authors**  **(year)** | **Number of participants (n) MS / HC** | **Sex ratio** | **Age in years MS / HC** | **EDSS** | **Disease duration** | **Disease course** | **Inclusion criteria** | **Exclusion criteria** |
|  |  |  |  |  |  |  |  | cognition, head injury with loss of consciousness for more than 30 minutes and a history of drug abuse |
| Belgium  **Ranchet et al. (43)** | 218 MS | 116 female (53%) | Pass: 52.41 (mean, SD 11.13)  Fail: 53.86 (mean, SD 10.95) | N/A | Pass: 11 years (median, Q1-Q3: 6-19), Fail: 14 years (median, Q1-Q3: 11-21) | N/A | Diagnosed MS | none |
| USA  *Raphail et al. (44)* | 31 MS | 24 (77%) female | 47.87 (mean, SD 9.81) | 5  (mean, SD 1.1, range 3.0 -6.5) | 11.86 years (mean, SD 9.93, range 0.58 – 38) | N/A | MS diagnosis, driver’s license for at least 1 year, actively driving with typical controls (i.e., without assistive devices such as additional hand/foot controls), EDSS or MSFC score | history of significant psychiatric or neurological condition other than MS, MS exacerbation in the last 90 days, simulator sickness |
| USA  **Schultheis et al. (45^*4^)** | 66 MS  30 HC | MS: 52 (79%) female | MS: 43.2 (mean, SD 8.07) | 3.42 (mean, range: 1.5-6.5) | 8.88 years (mean, SD 6.43) | RRMS: 57 (86%)  SPMS: 5 (8%) | MS: Diagnosed MS  All: active driver (1 driving occasion in a 1-month period in the past years), valid driver’s | All: history of other neurologic disease, clinical exacerbation in last month, psychiatric  illness, or substance |
| **Country**  **Authors**  **(year)** | **Number of participants (n) MS / HC** | **Sex ratio** | **Age in years MS / HC** | **EDSS** | **Disease duration** | **Disease course** | **Inclusion criteria** | **Exclusion criteria** |
|  |  | HC: 19 (63%) female | HC: 37.3 (mean, SD 10.33) |  |  | PPMS: 2 (3%)  Unknown: 2 (3%) | license, meet the minimum visual requirements (≥20/50 visual acuity in New Jersey or ≥20/40 in Pennsylvania), age ≥18 (Schultheis, 2010: 18-56) | abuse, medication that adversely affect cognition (steroids, benzodiazepines, neuroleptics, opioids, narcotic analgesics), <1 year driving experience, received driving rehabilitation or retraining were included, history of reckless driving and/or loss of driving privileges |
| USA  **Schultheis et al. (19^*4^)** | 66 MS | 52 (78.8%) female | 43.24 (mean, SD 8.07) | 3.41 (mean, range: 1.5-6.5) | 9.07 years (mean, SD 6.44) |  |  |  |
| USA  *Shawaryn et al. (16^*5^)* | 29 MS | 17 (59%) female | 43.3 (mean, SEM 1.6, range 21–55) | Ambulation index: 1.2 (SEM = .02; range 0-3) | 9.7 years (mean, SEM 1.4, range 1-22) years | RRMS: 14 (48%)  SPMS: 2 (7%)  Unknown: 13 (45 %) | MS: MS diagnosis  All: valid driver’s license, age < 55 years, active driving status, ≥ 1 year driving experience, ≥ 20/50 visual acuity rating in at least one eye (for New Jersey driver) or ≥20/40 visual acuity with or without corrective lenses for Pennsylvania, independent from the use of assistive driving devices | MS: exacerbation of symptoms within one month before testing, minimal to no physical limitations (>3 on the Ambulation Index) |
| USA  *Schultheis et al. (46^*5^)* | 28 MS  17 HC | With CI: 7 (54%) female  no CI: 10 (67%) female | With CI: 40.9 (mean, SEM 2.6)  no CI: 45.6 (mean, SEM 2.1) | N/A | With CI: 8.9 years (mean, SEM 1.8) | RRMS: 17 (61%)  SPMS: 2 (7%)  PPMS: 1 (4%) |  | HC: prior neurologic disorders, psychiatric illness, or history of substance abuse |
| **Country**  **Authors**  **(year)** | **Number of participants (n) MS / HC** | **Sex ratio** | **Age in years MS / HC** | **EDSS** | **Disease duration** | **Disease course** | **Inclusion criteria** | **Exclusion criteria** |
|  |  | HC: 13 (76%) female | HC: 43.8 (mean, SEM 2.0) |  | no CI:10.4 years (mean, SEM 2.2) | Unknown: 8 (28 %) |  |  |

MS = multiple sclerosis, HC = healthy controls, EDSS = Expanded Disability Status Scale, Pass = passing the on-road test, SD = standard deviation, Q1-3 = first quartile to third quartile, Fail = failing the on-road test, RRMS = relapsing remitting multiple sclerosis, MMSE = Mini-Mental State Exam, TG = training group, CG = control group, N/A = not available, PPMS = primary-progressive multiple sclerosis, SPMS = secondary-progressive multiple sclerosis, MATTIS DRS = MATTIS Dementia Rating Scale, MADRS = Montgomery and Asberg Depression Rating Scale, IQR = Interquartile range, FSS= the Fatigue Severity Scale, BDI-FS = the Beck Depression Index Fast Screen, PS = processing speed, EF = executive functions, MTO = Ministry of Transportation, MSFC = Multiple Sclerosis Functional Composite, SEM = standard error of the mean, CI = cognitive impairment
